# Differences in Adolescent Physical Fitness: A Multivariate Approach and Meta-analysis

**DOI:** 10.1007/s10519-015-9754-2

**Published:** 2015-10-19

**Authors:** Nienke M. Schutte, Ineke Nederend, James J. Hudziak, Eco J. C. de Geus, Meike Bartels

**Affiliations:** Department of Biological Psychology, Netherlands Twin Register, VU University Amsterdam, Van der Boechorststraat 1, 1081 BT Amsterdam, The Netherlands; EMGO+ Institute for Health and Care Research, VU University Medical Center, Van der Boechorststraat 7, 1081 BT Amsterdam, The Netherlands; Department of Psychiatry, Medicine, and Pediatrics, Vermont Center for Children, Youth and Families, University of Vermont, College of Medicine, 1 South Prospect, Burlington, VT 05401 USA

**Keywords:** Physical fitness, Twin study, Heritability, Exercise ability, Meta-analysis

## Abstract

Physical fitness can be defined as a set of components that determine exercise ability and influence performance in sports. This study investigates the genetic and environmental influences on individual differences in explosive leg strength (vertical jump), handgrip strength, balance, and flexibility (sit-and-reach) in 227 healthy monozygotic and dizygotic twin pairs and 38 of their singleton siblings (mean age 17.2 ± 1.2). Heritability estimates were 49 % (95 % CI 35–60 %) for vertical jump, 59 % (95 % CI 46–69 %) for handgrip strength, 38 % (95 % CI 22–52 %) for balance, and 77 % (95 % CI 69–83 %) for flexibility. In addition, a meta-analysis was performed on all twin studies in children, adolescents and young adults reporting heritability estimates for these phenotypes. Fifteen studies, including results from our own study, were meta-analyzed by computing the weighted average heritability. This showed that genetic factors explained most of the variance in vertical jump (62 %; 95 % CI 47–77 %, *N* = 874), handgrip strength (63 %; 95 % CI 47–73 %, *N* = 4516) and flexibility (50 %; 95 % CI 38–61 %, *N* = 1130) in children and young adults. For balance this was 35 % (95 % CI 19–51 %, *N* = 978). Finally, multivariate modeling showed that the phenotypic correlations between the phenotypes in current study (0.07 < *r* < 0.27) were mostly driven by genetic factors. It is concluded that genetic factors contribute significantly to the variance in muscle strength, flexibility and balance; factors that may play a key role in the individual differences in adolescent exercise ability and sports performance.

## Introduction

Physical fitness can be defined as a set of components that influences exercise ability and performance in sports (Caspersen et al. 1985). Because exercise ability may be a major driver of voluntary exercise behavior (Bryan et al. 2007; de Geus and de Moor 2008) it is important to understand the sources of variation in physical fitness. Studies on physical fitness have focused on maximal oxygen consumption, as aerobic fitness is a major determinant of exercise ability. However, exercise ability also entails muscular strength, flexibility, and motor control, all of which play an important role in health (Baranowski et al. 1992; Ortega et al. 2008). Several easy to perform tests exist that have been shown to provide reliable and valid indicators of these traits.

*Muscle strength* is defined as the maximal force that can be generated by a specific muscle or muscle group during a single movement. Measurements of muscle strength typically focus on the force generated by the elbow flexors or the knee extensors, typically at different angles of elbow flexion or knee extension. Strength can be measured with the muscle remaining at a fixed length (isometric) or while contracting (dynamic). The handgrip test, an easy and reliable measure, is by far the most commonly used measure for assessing isometric strength in epidemiological studies (Bohannon et al. 2011). For dynamic explosive strength, the vertical jump has been the most widely used test. *Balance* is a performance-related fitness component that relates to the maintenance of a stable body position (Caspersen et al. 1985) which is maintained by both sensory and motor systems (Tresch 2007). It can be measured using the Balance Error Scoring System (BESS) that is commonly used by researchers and clinicians and has a moderate to good reliability (Bell et al. 2011). *Flexibility*, defined as the ability of a specific muscle or muscle group to move freely through a full range of motion, can be assessed by the sit-and-reach test (reaching forward as far as possible from a seated position).

The two main factors that can influence individual differences in physical fitness are innate biological differences and environmental factors. The latter can be subdivided in influences shared with other members of the family (shared environmental influences) and person-specific or unique environmental influences, which includes error measurement but also comprises person specific exercise participation, training, and coaching. A design that is often used for partitioning total variance in genetic, shared environmental, and unique environmental components is the classical twin design. In a twin study, intrapair resemblance between two types of twin relationships is compared; genetically identical (monozygotic, MZ) and non-identical (dizygotic, DZ) twins. If the MZ resemblance for physical fitness is comparable to the DZ resemblance and non-zero, this constitutes evidence for shared environmental influences on the phenotype under study. If the MZ resemblance for physical fitness is higher than the DZ resemblance this constitutes evidence for genetic influences on the phenotype. Previous twin studies showed that genetic factors account for a substantial part of the variation in the aforementioned components of physical fitness.

Table 1 provides an overview of twin studies conducted in children, adolescents or young adults (published in English). It reports twin correlations and/or heritability estimates of similar or comparable components of physical fitness. Although the sample sizes are small for most studies, results consistently show moderate to high heritability estimates for vertical jump (ranging from 47 to 83 %) (Chatterjee and Das 1995; Kovar 1976; Maes et al. 1996). For the handgrip test, heritability estimates range from 32 to 77 % in children and young adults (Kovar 1976; Okuda et al. 2005; Silventoinen et al. 2008; Venerando and Milani-Comparetti 1970). Four studies report moderate heritability estimates for balance (24–46 %) (Maes et al. 1996; Vandenberg 1962; Williams and Gross 1980). Although the balance tests used were slightly different, all were indicators of static body balance ability. Finally, 18–55 % of the variation in flexibility (as measured by the sit-and-reach test) in children and young adults could be explained by genetic influences (Chatterjee and Das 1995; Maes et al. 1996; Okuda et al. 2005). In addition, the study by Maes et al. detected significant shared environmental influences on flexibility. Taken together, the existing studies confirm a role for genetic influences on the individual differences in physical fitness but estimates vary widely. This may reflect the modest sample sizes used in most studies, with one clear exception for handgrip (Silventoinen et al. 2008). Meta-analysis could be helpful to provide a more robust estimate of the heritability of components of physical fitness.

**Table 1:** Overview of heritability studies

Phenotype	Study	Sample	Age range	*r*_MZ_	*r*_DZ_	A (%)	C (%)	E (%)
Vertical jump	Kovar (1976)	17 MZ pairs	11–25			83		
		13 DZ pairs						
	Chatterjee and Das (1995)	30 MZ pairs	10–27	0.85	0.21	71		
		24 DZ pairs						
	Maes et al. (1996)	43 MZ pairs	10	0.65	0.28	47^a^/78^b^	–	53^a^/22^b^
		61 DZ pairs						
Handgrip	Venerando and Milani-Comparetti (1970)	24 MZ pairs	9–17			32		
		24 DZ pairs						
	Kovar (1976)	17 MZ pairs	11–25			63		
		13 DZ pairs						
	Okuda et al. (2005)	90 MZ pairs	10–14	0.78	0.49	77	–	23
		68 DZ pairs						
	Silventoinen et al. (2008)	1682 MZ pairs	16–25	0.66	0.35	66	3	31
		1864 DZ pairs						
Balance^c^	Vandenberg (1962)	40 MZ pairs	14–18			24		
		30 DZ pairs						
	Williams and Gross (1980)^d^	22 MZ pairs	11–18	0.51	0.33	27		73
		41 DZ pairs						
	Maes et al. (1996)	43 MZ pairs	10	0.46	0.32	46	–	54
		61 DZ pairs						
Flexibility^e^	Chatterjee and Das (1995)	30 MZ pairs	10–27	0.73	0.60	18		
		24 DZ pairs						
	Maes et al. (1996)	43 MZ pairs	10	0.84	0.54	38^a^/50^b^	32^a^/42^b^	30^a^/8^b^
		61 DZ pairs						
	Okuda et al. (2005)	90 MZ pairs	10–14	0.58	0.29	55		44
		68 DZ pairs						

Only unadjusted estimates are reported, except for Silventoinen et al. (2008) (age-adjusted results). A dash indicates that this component could be dropped from the model. If empty, only A was reported

^a^Males

^b^Females

^c^different balance tests, but all indicators of static body balance

^d^longitudinal study; only baseline results are shown here

^e^flexibility measured with the sit-and-reach test

A further theme that has not been extensively addressed is the extent of overlap between the genetic factors influencing these varied fitness phenotypes. Only a few studies provided some information on the (genetic) co-variation between components of physical fitness. Two studies reported moderate phenotypic correlations for muscle strength measures such as isometric strength measured by handgrip and knee extension. Multivariate modeling of these traits showed that part of the genes affecting muscle strength may be common to isometric strength measured by handgrip and knee extension (Silventoinen et al. 2008; Tiainen et al. 2004).

The four tests used in our study are derived from earlier work in fitness test batteries with the aim of constructing ‘unrelated’ components of health- and performance related fitness (Simons et al. 1969). Although Simons showed that these four fitness tests loaded on different factors, moderate phenotypic correlations (ranging from 0 to 0.54) between these tests were found in the 16–19 year olds. In a multivariate design it is possible to explore the source of covariance between these phenotypes. Information on the genetic association between various measures of physical fitness might be useful for meta-analyses over genetic association studies to examine the association of genetic variants with physical fitness.

To summarize, there is evidence for genetic influences on muscle strength (handgrip and vertical jump), balance, and flexibility but heritability estimates vary across samples. Multivariate genetic analyses on all four parameters have not been reported. To replicate and expand the literature on the genetic architecture of physical fitness components, we estimated the heritability of muscle strength measures (vertical jump and handgrip strength), balance and flexibility in a large sample of adolescent twins and their siblings and these estimates were incorporated in a meta-analysis on the heritability of muscle strength, flexibility and balance. Finally, in a multivariate design, the source of covariance among these fitness components was examined.

## Methods

### Participants

548 healthy adolescent twin pairs aged between 16 and 18, enrolled in longitudinal survey studies of the Netherlands Twin Register (van Beijsterveldt et al. 2013), were invited to participate in the study on the determinants of adolescent exercise behavior. Siblings of the twins within an age range of 12–25 years were also invited. Selection for invitation was based on the availability of longitudinal survey data on zygosity and regular leisure time exercise behavior. The aim was to have sufficient twins present from the entire spectrum of sedentary to vigorous leisure time exerciser and for each zygosity group. We started with a random selection, but if a zygosity group was underrepresented or if there were too little sedentary or vigorous exercisers, invitations were biased towards the underrepresented groups. In order to be eligible for the study, participants had to have no history of cardiovascular or respiratory disease, and being physically capable of engaging in exercise activities.

Participants were invited by sending a letter advertising the opportunity to test their fitness in addition to earning a gift voucher. All invitees had to be able and willing to visit the VU University in Amsterdam for lab testing. The final sample consisted of 227 complete twin pairs: 59 monozygotic male pairs (MZM), 36 dizygotic male pairs (DZM), 57 monozygotic female pairs (MZF), 42 dizygotic female pairs (DZF), 33 dizygotic opposite sex pairs (DOS) and 38 of their singleton siblings. Two additional sibling pairs participated (without a twin), resulting in a sample size of 498 participants. Mean age at time of the laboratory assessment was 17.2 ± 1.2.

All participants above 18 provided written consent and if the participants were under 18 consent was given by both of their parents/guardians and assent by the participant. All study procedures were reviewed and approved by the Medical Ethics Review Committee of the VU University Medical Center Amsterdam (NL35634.029.10).

### Components of physical fitness

On arrival at the laboratory, height and weight (Omron BF511, Omron Healthcare Europe B.V., The Netherlands) were measured. Subsequently, 4 fitness characteristics were examined: vertical jump, handgrip strength, balance, and flexibility.

#### Vertical jump

Explosive strength was measured with a vertical jump test that requires the participants to jump as high as possible, starting from a position of knee bending at a fixed knee angle immediately prior to the jump. Participants were instructed to jump straight up as much as possible and not go sideways. It was allowed to use the arms to help drive the body upwards. A successful jump was defined as one where at take-off the participants had the appropriate knee angle and landed their feet within a 10 cm radius of the start position. Jumping height was defined as the vertical displacement between of the trunk at the beginning and at the end of the jump measured by the displacement of a tapeline attached to the participants’ hip and a clipped to the floor. Best out of 3 jumps was documented (jumping height in centimeters).

#### Handgrip strength

Participants were instructed to hold a dynamometer (Baseline Digital Smedley Hand dynamometer, Fabrication Enterprises Inc., USA) in the dominant hand with arm at the side of the body and elbow at a 90° angle. When ready, the subject was encouraged to squeeze the dynamometer once with maximum effort (in kg), which should be maintained for about 5 s.

#### Balance

The Balance Error Scoring System (BESS) (Bell et al. 2011) was used to assess balance under 3 testing stances: double leg, single leg (non-dominant leg) and tandem (dominant foot in front of the non-dominant foot in heel-to-toe fashion, weight evenly distributed across both feet) on 2 surfaces (ground and foam pad). During the test, the eyes were closed and the hands were held on the hips. Each condition lasted for 20 s. We instructed the participants that if at any time they fell out of position, they were to return to the test position as quickly as possible. As the participants performed each 20-s trial, we observed and recorded the number of errors each subject made. An error was defined as opening eyes, lifting hands off hips, stepping, stumbling or falling out of position, lifting forefoot or heel, abducting the hip by more than 30°, or failing to return to the test position in less than 5 s. The total score was the total number of errors. For every participant, this number was recalculated (finals score was subtracted from 60) as such that a better balance was associated with a higher score.

#### Flexibility

Flexibility was measured using a standard sit-and-reach box (Baseline Sit-and-reach Trunk Flexibility Box, Fabrication Enterprises Inc., USA). Participants were instructed to sit on the floor with the legs fully extended and the soles of the feet flat against the box. One hand was placed on top of the other palms down. Then the subject reached forward along the measuring scale on the box as far as possible, without bending the knees. Best out of 3 reaches (in centimeters) was used for subsequent analyses.

### Genetic analyses

Genetic structural equation modeling in OpenMx (Boker et al. 2011) under R (R Development Core Team 2011) was used with the raw-data ML procedure for estimation of parameters. For all analyses, a threshold of *p* < 0.05 was considered for statistical significance. First, a so-called saturated model that estimated all parameters freely (a) was fitted to the data. Given the relative small sample size, with no power to test for sex-differences, and since (non-twin) siblings share, like DZ twins, on average 50 % of their genes, parameter estimates were constrained to be equal for males and females and for DZ twins and siblings. Main effects of sex and age and body mass index (BMI) on mean levels of components of physical fitness were considered in the model since these factors are associated with strength (Chatterjee and Chowdhuri 1991).

Cross-trait/cross-twin correlations and their 95 % confidence intervals were estimated for the MZ and DZ twins/siblings. Subsequently, 4 univariate models and a 4-variate Cholesky decomposition were fitted to the data to decompose the phenotypic statistics into sources of additive genetic variance/covariance (A), dominant genetic variance/covariance (D) or shared environmental variance/covariance (C) and unique environmental variance/covariance (E). Since C and D effects cannot be estimated simultaneously in the classical twin model, the ratio of the MZ correlations to the DZ correlations was used to determine which model (ACE or ADE) is most appropriate. Significance of variance–covariance components was tested by comparing the model including the specific component (e.g. ADE) to a model in which the component is constraint to be equal to zero (e.g. AE). The pattern of the factor loadings on the latent genetic and environmental factors in a Cholesky decomposition reveals a first insight into the etiology of covariances between the physical fitness components.

### Meta-analyses

In order to collect all studies on the heritability of the four components of physical fitness under study, a search of the electronic databases ISI Web of Knowledge and PubMed was conducted using *handgrip/muscle strength/vertical jump/explosive strength/flexibility/sit*-*and*-*reach/balance* and *genes/heritability/twin(s)* as key words. In addition, the reference lists of these articles were inspected. Articles (all-year) published in English and reporting twin correlations and/or heritability estimates of the vertical jump test, handgrip strength, balance and flexibility (sit-and-reach test) in a sample of children, adolescents and/or young adults up to the age of 30 were included, provided that these phenotypes were roughly comparable (i.e. protocol) to the phenotypes measured in the current study. These papers are shown in Table 1. For all studies, the univariate and unadjusted correlations and/or estimates were extracted, except for the study by Silventoinen et al. (2008) and Tiainen et al. (2004), who reported age-adjusted estimates only. While not all studies reported twin correlations, they did include an estimate of the heritability, therefore the meta-analyses were based on the heritability estimates. By weighing these heritability estimates from all studies by the number of participants, the weighted average heritability can be computed using Microsoft Excel (2010) (Li et al. 2003; Neyeloff et al. 2012). When the standard errors (SEs) or confidence intervals (CIs) of the heritability estimates were not reported, these were calculated using the SEs or CIs from studies who did report these statistics (Li et al. 2003). All studies reported one (equated) heritability estimate for males and females, except for Maes et al. (1996). These heritability estimates for males and females were treated if these were independent samples. Results from the current study were also included in the meta-analyses. For consistency, univariate models were fitted to our four phenotypes and the resulting heritability estimates were used in the meta-analyses. The *I*^*2*^ statistic was used to assess heterogeneity and was calculated as (*Q* − df)/*Q*, where *Q* is Cochran’s heterogeneity statistic and df the degrees of freedom (Higgins and Thompson 2002).

## Results

### Descriptives

Means and standard deviations for the fitness components of males and females are shown in Table 2. BMI (kg m^−1^) of this sample was (mean ± SD) 20.6 ± 2.5 for males and 21.8 ± 3.3 for females, comparable to the average 17 year olds in The Netherlands (Schonbeck et al. 2011). Males outperformed females for the vertical jump (*p* < 0.001) and handgrip (*p* < 0.001), whereas females performed better for balance (*p* < 0.001) and flexibility (*p* < 0.001). As expected, significant age effects were found on vertical jumping (*p* = 0.011) and handgrip (*p* < 0.001). Additionally, significant effects of BMI on mean levels of vertical jump (*p* = 0.026), handgrip (*p* = 0.041) and balance (*p* = 0.011) were detected. Because of significant sex, age, and BMI effects on the mean these factors were taken into account in further model fitting.

**Table 2:** Means and standard deviations of vertical jump, handgrip strength, balance and flexibility in males in females

	Males (*N* = 243)		Females (*N* = 255)	
	Mean	SD	Mean	SD
Vertical jump	45.8	6.4	35.5	5.4
Handgrip strength	40.2	8.0	29.5	4.7
Balance	45.1	6.6	47.1	6.6
Flexibility	19.8	10.1	29.0	9.7

Table 3 shows the phenotypic correlations (95 % confidence intervals) in the upper panel. Vertical jump was significantly associated with handgrip (0.27) and flexibility (0.10) but not with balance. Better balance was associated with higher scores on the handgrip (0.15) and flexibility test (0.10). No association between handgrip and flexibility was found. For all fitness components, the MZ correlation was higher than the DZ/sibling correlation (diagonal components of the lower two panels of Table 3), suggesting a genetic effect. For vertical jump, handgrip, and flexibility, the DZ/sibling correlations were less than half the MZ correlations, so shared environment factors were not further considered as a source of variance for these fitness parameters. Cross-twin/cross-trait correlations (off-diagonal correlations in Table 3) showed that in MZ twins handgrip was significantly associated with vertical jump (0.19) and balance (0.23), but not in DZ twins/siblings, suggesting a common set of genes influencing these components of physical fitness. Furthermore, flexibility was not significantly associated with vertical jump, handgrip and balance in MZ twins and DZ twins/siblings. The negative DZ twin/sibling correlations between handgrip strength and balance and handgrip strength and flexibility were not significant and most likely the result of a relatively small sample size.

**Table 3:** Phenotypic and cross-twin/cross-trait correlations (95 % CI) for vertical jump, handgrip strength, balance and flexibility estimated from the saturated model

	Vertical jump	Handgrip strength	Balance	Flexibility
Phenotypic correlations				
Vertical jump	1.00			
Handgrip strength	0.27 (0.18, 0.37)	1.00		
Balance	0.07 (−0.03, 0.16)	0.15 (0.05, 0.25)	1.00	
Flexibility	0.10 (0.01, 0.20)	0.08 (−0.02, 0.18)	0.10 (0.01, 0.19)	1.00
MZ correlations				
Vertical jump	0.53 (0.39, 0.64)			
Handgrip strength	0.19 (0.02, 0.36)	0.58 (0.44, 0.69)		
Balance	0.08 (−0.09, 0.25)	0.23 (0.06, 0.38)	0.34 (0.16, 0.49)	
Flexibility	0.10 (−0.07, 0.25)	0.10 (−0.06, 0.25)	0.03 (−0.13, 0.18)	0.79 (0.71, 0.85)
DZ/sibling correlations				
Vertical jump	0.20 (0.05, 0.34)			
Handgrip strength	0.07 (−0.07, 0.21)	0.27 (0.13, 0.40)		
Balance	0.04 (−0.10, 0.17)	−0.03 (−0.18, 0.11)	0.22 (0.07, 0.35)	
Flexibility	0.10 (−0.04, 0.24)	−0.12 (−0.27, 0.04)	0.09 (−0.06, 0.24)	0.31 (0.16, 0.44)

### Univariate results

Model fitting for vertical jump, handgrip and flexibility started with an ADE model. Dominant genetic influences were not significant (*p* > 0.05) and were dropped from the model. Heritability estimates were 49 % (95 % CI 35–60 %) for vertical jump, 59 % (95 % CI 46–69 %) for handgrip and 77 % (95 % CI 69–83 %) for flexibility. For balance, modeling started with an ACE model, as twin correlations for balance suggested the presence of shared environmental influences. However, C could be dropped from the model (*p* < 0.05). Genetic factors explained 38 % (95 % CI 22–52 %) of the variance in balance in our sample. The remaining variance in the four phenotypes was accounted for by unique environmental factors.

### Meta-analyses

The results of the meta-analyses are presented in Table 4. For vertical jump, results from four studies (the study by Maes et al. resulted in two sex-specific estimates) were used, including the current study. The heritability estimates of these studies are represented in the graph on the right as squares (with 95 % CIs) and the bottom square shows the weighted average heritability estimate of 62 % (95 % CI 47–77 %). This estimate falls within the CIs of all studies, except for the current study. The majority of variance in vertical jump in this combined child and young adult sample (*N* = 874) can be explained by genetic factors. Handgrip measured in a combined sample of 9–25 year olds (*N* = 4516) showed a weighted average heritability estimate of 63 % (95 % CI 47–73 %). This estimate falls within the CIs of all studies included in this meta-analysis. For balance, a weighted average heritability estimate of 35 % (95 % CI 20–41 %, *N* = 1704) was found, which falls perfectly in the CIs of the included studies. Four studies reported a heritability estimate for flexibility in children and young adults, ranging from 18 to 77 % (current study). The meta-analytic weighted average heritability of 50 % (%95 CI 38–61 %, *N* = 1130) falls outside the CIs of these two heritability estimates. The meta-analyses for handgrip and balance showed low heterogeneity (*I*^2^ = 18 and 0 %). However, high *I*^2^ values were detected for vertical jump and flexibility (46 and 94 %), suggesting that differences in studies are not caused by sampling error only.

**Table 4:** Heritability estimates (95 % CI) of the studies used in the meta-analyses

Phenotype	Study	Sample Size	Heritability	
Vertical Jump	Kovar (1976)	60	83 (47, 119)	
	Chatterjee and Das (1995)	108	71 (44, 98)	
	Maes et al. (1996)^a^	105	47 (20, 74)	
	Maes et al. (1996)^b^	103	78 (51, 106)	
	Current study	498	49 (35, 60)	
	**Meta-analysis**	**874**	**62 (47, 77)**	
Handgrip	Venerando and Milani-Comparetti (1970)	96	32 (−6, 70)	
	Kovar (1976)	60	63 (15, 111)	
	Okuda et al. (2005)	316	77 (56, 98)	
	Silventoinen et al. (2008)	3546	66 (60, 72)	
	Current study	498	59 (46, 69)	
	**Meta-analysis**	**4516**	**63 (47, 73)**	
Balance	Vandenberg (1962)	146	24 (−4, 52)	
	Williams and Gross (1980)	126	27 (−3, 57)	
	Maes et al. (1996)	208	46 (23, 69)	
	Current study	498	38 (22, 52)	
	**Meta-analysis**	**978**	**35 (19, 51)**	
Flexibility	Chatterjee and Das (1995)	108	18 (3, 33)	
	Maes et al. (1996)^a^	105	38 (23, 53)	
	Maes et al. (1996)^b^	103	50 (35, 65)	
	Okuda et al. (2005)	316	55 (46, 64)	
	Current study	498	77 (69, 83)	
	**Meta-analysis**	**1130**	**50 (38, 61)**	

In bold the weighted average heritability estimate (95 % CI)

All confidence intervals are calculated based on sample size

^a^Males

^b^Females

### Multivariate analyses

Based on the overall correlational structure, multivariate model fitting was started with an ADE model. Dominant genetic influences were not significant (*p* = 0.659). Standardized components from this final model for additive genetic and unique environmental influences on the four components of physical fitness and their covariances are presented in Table 5. The diagonals in the upper panel show the heritability estimates for the four phenotypes from the multivariate model. The off-diagonal values show that the majority of the phenotypic correlations between the phenotypes under study could be explained by genetic factors (74–99 %) except for vertical jump and flexibility, of which environmental factors explained more than half of the phenotypic correlation (53 %). Significant genetic correlations were found for handgrip and vertical jump (*r*_G_ = 0.46, 95 % CI 0.27–0.65), handgrip and balance (*r*_G_ = 0.32, 95 % CI 0.11–0.52) and balance and flexibility (*r*_G_ = 0.18, 95 % CI 0.01–0.37). In addition, a significant environmental correlation was found between vertical jump and flexibility (*r*_E_ = 0.22, 95 % CI 0.04–0.38).

**Table 5:** Standardized estimates (95 % CI) for additive genetic (A) and unique environmental influences (E) on the four components of physical fitness and their covariance based on the full AE Cholesky model

	Vertical jump	Handgrip strength	Balance	Flexibility
A				
Vertical jump	0.49 (0.36, 0.61)			
Handgrip strength	0.85 (0.55, 1)	0.60 (0.48, 0.70)		
Balance	0.83 (0, 1)	0.99 (0.49, 1)	0.39 (0.23, 0.52)	
Flexibility	0.47 (0, 0.89)	0.94 (0.64, 1)	0.74 (0.09, 1)	0.78 (0.70, 0.83)
E				
Vertical jump	0.51 (0.39, 0.64)			
Handgrip strength	0.15 (0, 0.45)	0.40 (0.30, 0.52)		
Balance	0.17 (0, 1)	0.01 (0, 0.51)	0.61 (0.48, 0.77)	
Flexibility	0.53 (0.11, 1)	0.06 (0, 1)	0.26 (0, 0.91)	0.22 (0.17, 0.30)

## Discussion

To examine the heritability of and genetic co-variation between various components of physical fitness, genetic models were fit to data from 498 late-adolescent twins and their siblings. Univariate modeling showed that a moderate to large part of the individual differences in components of physical fitness is accounted for by genetic differences between individuals. The remaining variance was accounted for by unique environmental effects. Muscle strength, flexibility, and balance all contribute to exercise ability and performance in sports (Caspersen et al. 1985; Gleim and McHugh 1997; Hrysomallis 2011; Ruiz et al. 2006). Strength and flexibility are not only performance-related but also health-related (Baranowski et al. 1992; Caspersen et al. 1985). Lower levels of these components measured in childhood and adolescence are associated with cardiovascular risk factors, such as hypercholesterolemia or hypertension in adulthood (Ortega et al. 2008; Wedderkopp et al. 2003). For instance, data from the AVENA study showed an association of lower scores of maximal handgrip and explosive strength in adolescent females and a cardiovascular risk score (Garcia-Artero et al. 2007). Balance, on the other hand, is considered mainly a performance-related fitness component (Caspersen et al. 1985). It showed a moderate heritability estimate of 38 % compared to strength and flexibility, demonstrating that most of the variance can be explained by person-specific environmental factors. These findings confirm findings by Maes et al. (1996) that components of physical fitness that are only performance-related are less under genetic control than components that are both performance and health-related.

The heritability estimates found in the current study were confirmed in meta-analyses of all studies reporting on the heritability of these phenotypes in twin samples under age 30 with some notable differences. For balance, all studies, including the current study, report more or less similar heritability estimates (24–46 %) and showed a rather homogenous picture, whereas the meta-analysis of flexibility showed heterogeneity as two out of five studies report an estimate significantly lower (Chatterjee and Das 1995) or higher (current study) than the meta-analytic heritability estimate of 50 %. A source of this variation might be the age of the subjects as Okuda et al. and Maes et al. measured flexibility in children and reported a lower heritability compared to the current study in late-adolescents. Chatterjee and Das measured flexibility in subjects with a much wider age range (10–27) and found a heritabilty estimate of only 18 %. However, after adjustment for age, this estimate increased to 50 % (Chatterjee and Das 1995). For muscle strength meta-analyses resulted in weighted average heritability estimates of 62 % for vertical jump and 63 % for handgrip strength. This estimate generally fell within the confidence intervals of all the studies, despite the wide range of heritability estimates of the included studies (47–83 % for vertical jump and 32–77 % for handgrip). Our study, however, reports a 13 % lower heritability for vertical jump. Taken together, from the analyses presented we conclude that at least half of the variance in vertical jump, handgrip strength and flexibility and a substantial part of the variance in balance in children and young adults (<30 year) can be explained by genetic factors.

Environmental factors that are shared by the twins (such as the family environment) do not seem to play a major role in explaining individual differences in physical fitness components in our late-adolescent sample. As the correlations for DZ twins/siblings were low and non-significant, shared environment factors were not further considered as a source of variance for these fitness parameters. This does not rule out a small contribution of shared environmental influences, as this is hard to detect in samples of this size, even if the power to detect shared environmental influences was increased by adding siblings to the design. Of interest, twin studies on voluntary exercise behavior show that the influence of these shared environmental factors is significant at young ages, but decreases or has completely disappeared when reaching adolescence (Huppertz et al. 2012; Stubbe et al. 2005; van der Aa et al. 2010). Twin correlations in Table 1 do suggest the presence of shared environmental influences as the DZ/sibling correlations for handgrip, balance and flexibility were higher than half the MZ correlations. In the current study, the DZ/sibling correlation for balance was higher than half the MZ correlation. Only two studies (Maes et al. 1996; Silventoinen et al. 2008) reported a significant contribution of shared environmental factors. Posthuma and Boomsma (2000) showed that to detect shared environmental factors with a power of 80 %, a sample size of more than 2000 individuals is needed (extended twin design) to detect shared environmental influences (when A = 20–50 % and C = 10–20 %). A very large study on handgrip estimated C at 3 % (Silventoinen et al. 2008) in adolescents and young adults. Our sample size, and even most of those accrued in the meta-analyses were too small to detect such small C effects (Posthuma and Boomsma 2000). Of note, when C is dropped from the model, resemblance between the twin and co-twin/sibling will be modeled as A. As a result, the variance that is attributed to genetic factors might be slightly overestimated (with smaller 95 % CIs) in small samples, which, in turn, might have biased our meta-analytic heritability estimate.

In the current study, some of the physical fitness components were moderately, but significantly, associated to each other although they reflect different dimensions of physical fitness (Simons et al. 1969). These cross-trait associations are mostly driven by genetic factors (the association between vertical jump and handgrip strength and between handgrip strength and balance) or unique environmental factors (association between vertical jump and flexibility). Silventoinen et al. (2008) reported genetic correlations of 0.43 up to 0.54 for handgrip, knee extension and elbow flexion. In addition, Tiainen et al. (2004) showed that that handgrip and knee extension strength are measures under the control of the same genetic component. Furthermore, high genetic correlations (0.62–0.91) were reported for maximal isometric, concentric and eccentric muscle strength and muscle cross-sectional area of the elbow flexors (De Mars et al. 2007). Genetic correlations in our sample ranged from 0.18 to 0.46. The genetic overlap between vertical jump strength and handgrip strength in our sample can be explained from a muscle biology viewpoint, as both explosive and isometric strength are dependent on the cross-sectional area of the contributing muscles. The specific genetic factors contributing to vertical jump might entail muscle coordination strategy, the percentage of type II fibers and elastic components.

A major future challenge is to identify the specific genes underlying the heritability of these four components of physical fitness. Candidate genes studied have focused on insulin-like growth factor- and myostatin-related genes and genes involved in inflammatory factors. Linkage analyses revealed several additional regions of interest in the genome, although individual genes could not be identified as yet (see Thomis and Aerssens 2012 for a review). One of the most studied polymorphisms is the R577X variation in the *ACTN3* gene. This gene seems to influence the performance of fast skeletal muscle fibers and *ACTN3* XX homozygotes may have modestly lower skeletal muscle strength in comparison with R-allele carriers (Yang et al. 2003). No large-scale genome-wide association (GWA) studies have been conducted on these phenotypes, which has proven to be a successful approach to understanding the heritability of many health-related risk factors and disease (Flint 2013; Visscher et al. 2012). This is unfortunate, because the components of physical fitness used in this study are relatively easy to measure (compared to for example maximal oxygen consumption) in large samples and show substantial heritability, suggesting that a GWA meta-analysis effort could be successful. Moreover, the moderate but significant genetic association between handgrip and vertical jump suggests that meta-analysis over genetic association studies that use comparable traits is valid, and that the traits do not need to be exactly similar to capture the latent genetic factors.

Some limitations must be considered while interpreting our results. An important assumption underlying twin studies is that twins are fully representative compared to the general population. Silventoinen et al. reported that singletons showed extra variation in weight and strength measured compared to twins, which could lead to inflated heritability estimates (Silventoinen et al. 2008). Furthermore, the siblings in our study had a very wide age range (12–25) which may be a problem as the younger siblings may still be pubertal, compared to the rest of the subjects. Inter-individual variation in maturation is an established factor that affects strength and power. However, when we tested for possible effects of these maturational differences between the twins and younger siblings by repeating the analysis with a restriction on age (no siblings) comparable results emerged (data not shown). The meta-analyses for vertical jump and flexibility showed moderate to high heterogeneity, indicating that differences between studies are not caused by sampling error only, but may also reflect population-specific differences. Although we aimed for including only studies in which fitness tests conceptually measured the same phenotype, differences in testing procedures might also add to the heterogeneity. In addition, it may be argued that in our meta-analyses it might not be justified to compare samples with different age ranges, due to differences in biological maturity in children and young adults. However, there are limited studies on the heritability of these components of physical fitness and combining the samples will increase power. Moreover, most studies presented in Table 1 had sample sizes too small to detect or account for gender differences, therefore gender differences were not taken into account when performing the meta-analyses. Finally, whereas we aimed to standardize the protocol as much as possible, differences in leg-muscle warm up and back stretching might partly explain the significant environmental association between vertical jump and flexibility.

To summarize, the analyses performed in this study confirm a significant contribution of genetic factors to the four physical fitness components and to their association. Understanding the genetic basis of fitness parameters may help us to understand the individual differences in regular voluntary exercise behavior, which show substantial heritability, particularly at the end of adolescence (Huppertz et al. 2012). Individual differences in muscle strength and flexibility co-determine late-adolescent exercise ability (Caspersen et al. 1985; Gleim and McHugh 1997; Hrysomallis 2011; Ruiz et al. 2006). Above-average exercise ability will allow an individual to gain more in exercise performance than others and this may lead to enhanced feelings of competence in exercise and sports. These rewarding effects will support longer term maintenance of exercise behavior. Vice versa, aversive mood effects which could be induced by below-average performance have been found to predict drop out from an exercise program (Williams et al. 2008). Physical fitness can be therefore a major driver of voluntary exercise behavior (Bryan et al. 2007; de Geus and de Moor 2008). Increased efforts to unravel the molecular genetic pathways underlying the heritability of fitness parameters are direly needed.
